# TNF-α blockade suppresses pericystic inflammation following anthelmintic treatment in porcine neurocysticercosis

**DOI:** 10.1371/journal.pntd.0006059

**Published:** 2017-11-30

**Authors:** Siddhartha Mahanty, Miguel A. Orrego, Carla Cangalaya, M. Paz Adrianzen, Gianfranco Arroyo, Juan Calcina, Armando E. Gonzalez, Héctor H. García, Cristina Guerra-Giraldez, Theodore E. Nash, for the Cysticercosis Working Group in Peru

**Affiliations:** 1 Laboratory of Parasitic Diseases, National Institute of Allergy and Infectious Diseases, National Institutes of Health, Bethesda, Maryland, United States of America; 2 Laboratorio de Inmunopatología en Neurocisticercosis, Facultad de Ciencias y Filosofía, Universidad Peruana Cayetano Heredia, Lima, Peru; 3 Facultad de Medicina Veterinaria, Universidad Nacional Mayor de San Marcos, Lima, Peru; 4 Unidad de Cisticercosis, Instituto Nacional de Ciencias Neurológicas, Lima, Peru; Universidade Federal de Minas Gerais, BRAZIL

## Abstract

**Background:**

Neurocysticercosis (NCC) is an infection of the brain with the larval cyst of the tapeworm, *Taenia solium*. Cysticidal treatment induces parasite killing resulting in a post inflammatory response and seizures, which generally requires corticosteroid treatment to control inflammation. The nature of this response and how to best control it is unclear. We investigated the anti-inflammatory effects of pretreatment with etanercept (ETN), an anti-tumor necrosis factor agent, or dexamethasone (DEX), a high potency corticosteroid, on the post treatment inflammatory response in naturally infected pigs with neurocysticercosis after a single dose of the cysticidal drug praziquantel (PZQ).

**Methodology/Principal findings:**

We followed the methods from a previously developed treatment model of NCC in naturally infected swine. The four study groups of infected pigs included 3 groups treated with PZQ on day 0: PZQ-treated alone (100 mg/kg PO; n = 9), pretreated with dexamethasone (DEX, 0.2 mg/kg IM administered on days -1, +1 and +3; n = 6), and pretreated with etanercept (ETN, 25 mg IM per animal on days -7 and 0; n = 6). The fourth group remained untreated (n = 3). As measured by quantitative RT-PCR, ETN pretreatment depressed transcription of a wide range of proinflammatory, regulatory and matrix protease encoding genes at 120 hr post PZQ treatment in capsules of cysts that demonstrated extravasated Evans Blue (EB) (a measure of blood brain barrier dysfunction) compared to animals not receiving ETN. Transcription was significantly depressed for the proinflammatory genes tumor necrosis factor (TNF)-α, and interferon (IFN)-γ; the inflammation regulating genes cytotoxic T-lymphocyte-associated protein (CTLA)4, interleukin (IL)-13 and transforming growth factor (TGF)-β; the tissue remodeling genes matrix metalloprotease (MMP)1 and 9, tissue inhibitors of metalloproteases (TIMP)1 and 2, and the genes regulating endothelial function vascular endothelial growth factor (VEGF)1, angiopoietin (Ang)1, Ang 2, and platelet endothelial cell adhesion molecule (PECAM)-1. In contrast, transcription was only modestly decreased in the DEX pretreated pigs compared to PZQ alone, and only for TNF-α, IL-6, IFN-γ, TGF-β and Ang1. IL-10 was not affected by either ETN or DEX pretreatments. The degree of inflammation, assessed by semi-quantitative inflammatory scores, was modestly decreased in both ETN and DEX pretreated animals compared to PZQ treated pigs whereas cyst damage scores were moderately decreased only in cysts from DEX pretreated pigs. However, the proportion of cysts with EB extravasation was not significantly changed in ETN and DEX pretreated groups.

**Conclusions/Significance:**

Overall, TNF-α blockade using ETN treatment modulated expression of a large variety of genes that play a role in induction and control of inflammation and structural changes. In contrast the number of inflammatory cells was only moderately decreased suggesting weaker effects on cell migration into the inflammatory capsules surrounding cysts than on release of modulatory molecules. Taken together, these data suggest that TNF-α blockade may provide a viable strategy to manage post-treatment pericystic inflammation that follows antiparasitic therapy for neurocysticercosis.

## Author summary

Infection of the brain with larvae of the tapeworm *Taenia solium* is called neurocysticercosis (NCC), a disease with varied and serious neurological symptoms. Therapy requires antiparasitic drugs and corticosteroids to prevent seizures caused by treatment due to inflammation around dying parasites. The gene expression of the proinflammatory molecule tumor necrosis factor alpha (TNF-α) is increased in NCC. We treated three groups of naturally infected pigs with an antiparasitic drug: one group was also pretreated with an anti-TNF-α inhibitor, the second one with a corticosteroid, and the third was not pretreated. All pigs were infused with Evans blue dye (EB), which leaks where the blood brain barrier is damaged by inflammation around cysts. We compared the expression of several genes involved in inflammation, healing and fibrosis and regulation of vascular function in tissues surrounding cysts. In inflamed samples showing leaked EB, the inhibition of TNF-α suppressed nearly all the genes assessed, and this suppression was significantly stronger than the moderate decrease caused by corticosteroid pretreatment on most of the genes. On microscopic examination, the inflammation observed was slightly decreased with both pretreatments in relation to the group that was not pretreated. We believe that the inflammatory route that includes TNF-α should be further explored in the search for better management of inflammation directed to degenerating cysts.

## Introduction

Neurocysticercosis (NCC), an infection of the central nervous system (CNS) by the larval stage (cysticercus) of the parasitic cestode *Taenia solium*, is a major cause of epilepsy in developing countries and a serious public health burden [1–4]. The disease is endemic to regions across the world where pigs are raised and allowed to roam freely with access to human waste [1, 5]. The occurrence of seizures and other symptoms of NCC depend on the number, location and distribution of cysticerci, the intensity of brain inflammation and the degenerative stage of the parasite, resulting in a wide variety of manifestations [2, 6].

A notable feature of *T*. *solium* infections is that viable cysts provoke minimal or no host-directed inflammatory responses. However, degenerating cysts or cysts damaged by anthelmintic treatment provoke inflammatory responses that can have pathological consequences on brain tissues surrounding the dying parasite [2, 5, 7]. Consequently, inflammation around degenerating cysts in the brain parenchyma generally results in seizures, whereas inflammation in the subarachnoid spaces causes diffuse and/or focal arachnoiditis frequently resulting in hydrocephalus, infarctions and nerve entrapments. Cysts in the ventricles commonly cause hydrocephalus due to mechanical obstruction of cerebrospinal fluid (CSF) outflow or to ventriculitis and scarring [1, 8].

The pathological inflammatory response induced by cysticidal drugs can interfere with treatment. Although corticosteroids are almost universally used to suppress inflammation and control symptoms, the ideal regimen for the safe and effective use of corticosteroids or other anti-inflammatory agents in multicystic or complicated NCC has not been determined. As a result, the dose, duration and type of corticosteroid used are frequently based on the individual practitioner’s experience or preference [5]. A better understanding of the acute inflammatory responses induced by treatment is necessary to formulate simple, safe and more effective treatment measures.

Studies of human and animal models of NCC indicate that inflammatory mediators produced by innate and adaptive immune cells play an important role in regulating inflammation both locally and systemically [9–16]. We previously demonstrated that expression of mediators of inflammation such as tumor necrosis factor (TNF)-α, interleukin (IL)-6 and interferon (IFN)-γ was up regulated following anthelmintic treatment around cysts that displayed disruption of blood brain barrier integrity [17]. These findings suggested points of attack to suppress specific pathways controlling treatment-induced inflammation to avoid the serious adverse effects of global immunosuppression associated with corticosteroids.

In the present study we focused on the TNF-α pathway of inflammation because of its importance in this infection. Changes in expression of genes encoding a number of inflammatory mediators and regulatory factors following treatment with praziquantel were determined in pericystic brain tissue from infected pigs following blockade of TNF-α with etanercept (ETN), a competitive inhibitor of TNF-α, and compared to corresponding tissues from a group of PZQ-treated pigs pretreated with corticosteroids and a control group of PZQ-treated pigs who did not receive any pretreatment.

## Methods

### Study animals

**Fig 1:**
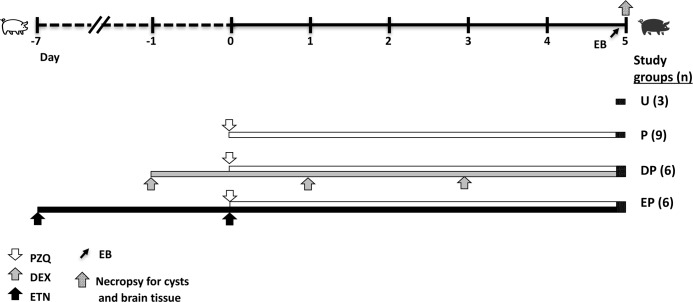
Schematic outline of the schedule of treatment with anti-inflammatory agents and praziquantel (PZQ), showing number of pigs per group and necropsy time points when cyst and brain samples were collected for histopathology and analysis of gene expression by quantitative RT-PCR. The experimental groups correspond to those described in detail in Methods. EB: Evans blue; PZQ: praziquantel 100 mg/kg PO, on day 0; DEX: dexamethasone 0.2 mg/kg IM, on days –1, +1 and +3; ETN: etanercept 25 mg/pig IM on days –7 and 0.

Twenty-four *T*. *solium*-infected outbred pigs, confirmed by a positive tongue examination for cysts, were obtained in Huancayo, Peru, a town in a region of Peru endemic for cysticercosis. Four healthy outbred uninfected pigs purchased in Lima, Peru served as a source of tissues to normalize the gene expression assays; they did not receive any treatment. The four study groups included: untreated (U), anthelmintic treatment with praziquantel (PZQ, 100 mg/kg; P), dexamethasone (DEX, and PZQ; DP) and etanercept (ETN and PZQ; EP). The experimental design, including treatment and sample collection schedule is shown in Fig 1. Pigs were housed in the animal facility of the San Marcos Veterinary School. A hundred and twenty hours after administration of PZQ, the pigs were anesthetized with ketamine (10 mg/kg, intramuscular injection) and xylazine (2 mg/kg, both from Agrovetmarket SA, Peru), for an intravenous catheterization and infusion of Evans Blue (EB) and euthanized with sodium pentobarbital (25 mg/kg kg every 30 min for two hours, intravenous injection; Montana SA, Peru).

### Ethics statement

The study protocol and procedures were reviewed and approved by the Comité Institucional de Ética para el Uso de Animales–CIEA (Institutional Ethics Commitee for the Use of Animals) of the Veterinary School of San Marcos University in Lima, Peru (Protocol numbers 006 for Universidad Nacional Mayor de San Marcos and 62392 for Universidad Peruana Cayetano Heredia). The Comité is registered in the Office for the Wellbeing of Laboratory Animals of the Department of Health and Human Services of the National Institutes of Health with Policy Number A5146-01. All procedures used at the Veterinary Medicine Faculty of Universidad National Mayor de San Marcos (FMV-UNMSM) adhere to the International Guiding Principles for Biomedical Research that Imply the Use of Animals by the Council of International Organizations of Medical Sciences (CIOMS), Geneva, 1985.

### Treatment conditions and EB infusion

As shown in Fig 1, infected pigs were treated with a single dose of praziquantel (PZQ, 100 mg/kg PO, on day 0; Montana SA, Peru), pretreated with DEX (0.2 mg/kg IM, on days -1, +1 and +3; Química Farvet, Mexico), ETN (25 mg/pig on days -7 and day 0, when PZQ treatment was also administered; Amgen, CA) or no drugs and sacrificed 120h later (n = 21, total). Three untreated infected pigs were used as controls. Two hours before euthanasia, pigs were anesthetized and infused with 2% EB (80 mg/kg; Sigma-Aldrich, St. Louis, MO) in saline solution (NaCl 0.85%, Laboratories Baxter, Colombia, Baxter del Peru) by intravenous injection via the carotid artery. Just after euthanasia, the pigs were perfused with chilled saline solution containing heparin (NaCl 0.85% with 10 U of heparin/mL) and the brains were immediately removed to collect specimens.

### Collection and selection of specimens for qPCR and histology

Brains were sliced into 10-mm thick sections on dry ice. The presence of unambiguously blue and clear stained cysts and tissues was documented by gross examination. Tissues surrounding visible cysts (pericystic “capsules”, histologically consisting of collagen and cellular infiltrate) were sampled from each brain (~1 mL fragments) and either placed in RNALater solution (Invitrogen, Gaithersberg, MD) for RNA extraction or fixed, together with the cyst, in 10% formalin (PBS pH 7.2 with 3.7% formaldehyde) for histological examination. More cysts were selected for histology than for RNA studies because of relatively limited resources for the latter (See S1 Table).

Paraffin sections were processed using standard procedures and stained with hematoxylin and eosin (HE) and Masson’s Trichrome stain (MT). Only capsules containing a whole cyst or a cyst wall surrounded by host tissue were selected for histological examination. We focused on the blue cysts capsules for qPCR analysis for two main reasons. Firstly, our previous studies had shown that significant upregulation of inflammatory and regulatory genes was apparent in blue capsules and not in clear capsules [17, 18]. Secondly, the proportion and number of clear capsules following PZQ treatment was dramatically reduced, resulting in unacceptably large variance in the parameters studied and making statistical inference unreliable or not possible (See S2 Table).

### Histological examination

Histological examination and analysis were performed exactly as reported previously [17, 18]. Briefly, a low power image of a section of whole cyst was first examined to assess the circumferential proportion associated with pericystic inflammation. Higher power examination was then employed to determine the proportion of the cyst circumference showing each inflammatory stage (IS) present around each cyst. The classification of the inflammatory stages followed the schema described by Álvarez et al [19] and Londoño et al [20], and adapted by us in a previous study [18]. According to this scheme, stage categorization of the inflammatory infiltrate was semi-quantitatively determined based on the average number of cells per high power field, and the thickness and location of type I collagen fibers around the cysts and in pericystic capsules [17]. Using these measurements, IS 1 to 4 represented increasing severity of inflammatory reaction and pathology. Similarly, cyst wall damage was categorized into four stages (Damage Score: DS0-DS3 by severity of tissue disruption in the cysts, as outlined by Londoño et al. [20]. Composite inflammatory (IS) and damage scores (DS) were determined for each cyst using the formula: Composite IS or DS score = Sum [(Score x % of cyst circumference) x 100]. The percentage of circumference was rounded off to increments of 25% (i.e., 0, 25, 50, 75 or 100%). IS and CD scores from the untreated (U) and PZQ-treated (P) groups pooled data from the experimental groups in the current experiments and with those from previous experiments of identical design published elsewhere [17].

### Generation of cDNA and qPCR

qPCR was performed on subsets of cysts selected from cysts with unambiguously clear or blue capsules. The distribution of cysts from each experimental group is shown in S1 Table. Fragments of brain tissue (50–100mg) containing only cyst capsules (no cyst) were homogenized in 1 ml TRIzol (Invitrogen, Gaithersberg, MD) for standard RNA chloroform extraction. cDNA was generated from 1 μg of total RNA using the High-Capacity cDNA Reverse Transcription Kit with multiscribe RT polymerase and random primers (Applied Biosystems, Foster City, CA) in 20-μl reactions by incubation for 10 min at 25°C followed by 60 min at 37°C, 5 min at 95°C on a thermocycler (MJ Research PTRC-200, BioRad-MJ Research, Hercules, CA). Real time PCR (qPCR) was performed in 10-μl reaction volumes using the Taqman Gene Amplification System (Applied Biosystems) with commercially available primer-probe combinations using conditions recommended by the manufacturer. We used 18S rRNA as a control gene to validate RNA integrity and primer probe pairs for porcine TNF-α, IL-6, IFN-γ, IL-10, CTLA4, IL-13, TGF-β, MMP1, MMP9, TIMP1, TIMP2, VEGF, PECAM1, Ang1 and Ang2 genes. qPCR reactions, run in triplicate, used the following cycling parameters: preincubation of 2 min at 50°C and 10 min at 95°C followed by 40 cycles of 15 sec at 95°C and 1 min at 60°C, on an AB StepPlusOne cycler (Life Technologies, Grand Island, NY). Results for each gene were expressed as relative to the expression of 18S rRNA using the X-fold value defined by the 2^-ΔΔCT^ formula [21]. The number of cysts analyzed for each marker differed within a given experimental group, because limitations on the amount of RNA extracted prevented us from analyzing each cyst for all the desired markers.

### Statistical analysis

Non-parametric statistics, Mann-Whitney U test for two groups and Kruskal-Wallis test for multiple groups, were calculated using Prism® software (Graphpad, San Diego, CA) for comparisons of the above parameters between infected pigs that were not treated or treated with one of the two anti-inflammatory agents (PZQ or ETN), and between clear cysts and those with EB staining (blue cysts). Corrections for multiple comparisons were applied for pairwise comparisons. Differences with p-values of <0.05 were considered statistically significant. Two-way contingency analysis, for example, comparing differences in proportions of cysts between PZQ-treated and PZQ plus DEX-pretreated pigs were performed by the Fisher’s exact test.

## Results

### Animal characteristics and histopathological observations

**Fig 2:**
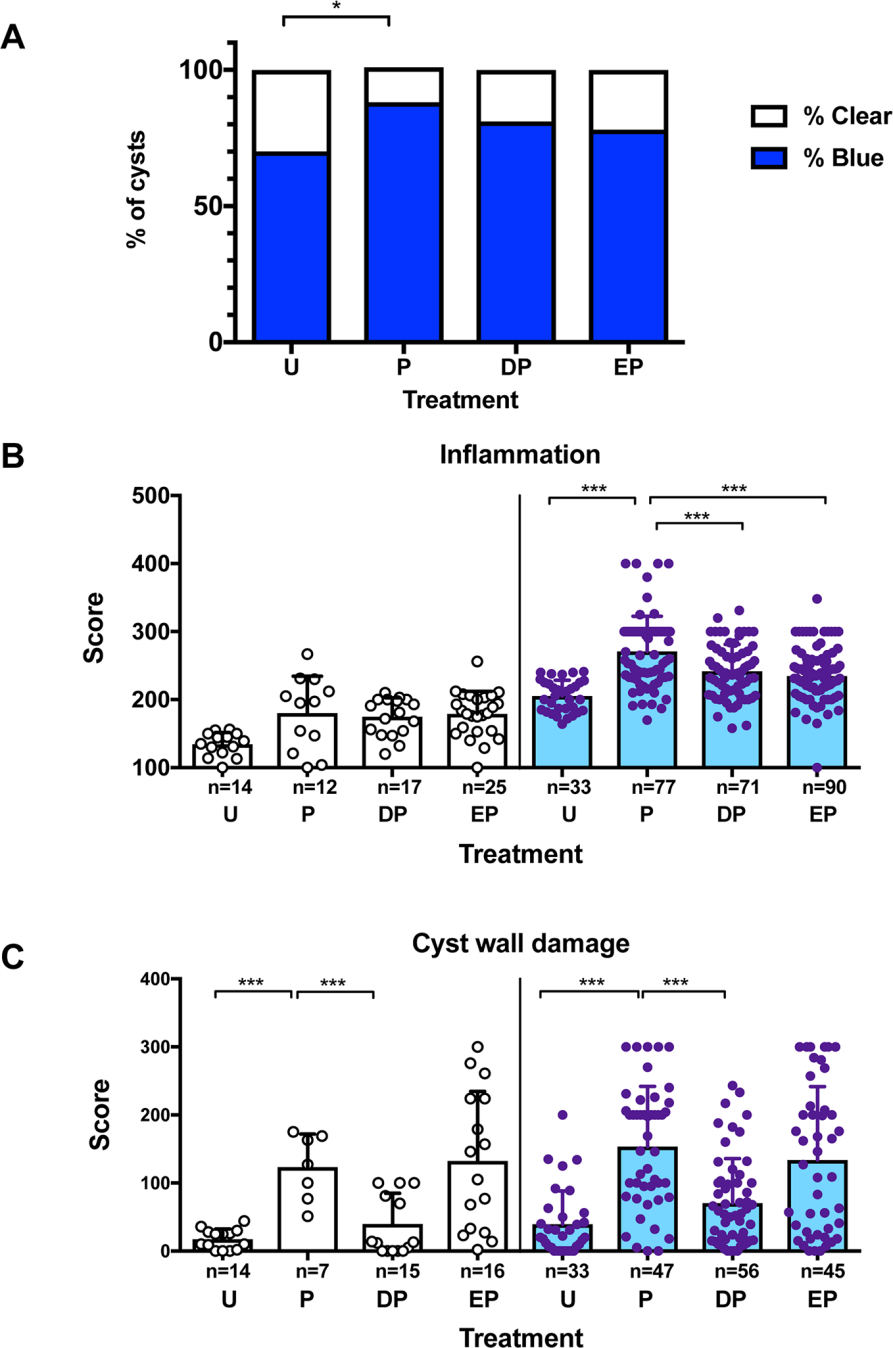
Vascular leakage, inflammatory infiltration and cyst wall damage following PZQ treatment with or without pretreatment with anti-inflammatory agents in naturally infected pigs. **A**. The proportion of cysts with extravasation of EB dye as described in Methods and Fig 1. Each bar represents the total (100%) of all cysts examined in the group labeled in the X-axis, and the shaded portion represents the proportion with EB in the cyst capsules., **B**. Inflammation scores (IS) in cyst capsules recovered from pigs in the treatment groups (see Methods for description of scores). Each dot represents an individual cyst capsule, and bars indicate means ± standard deviations for the group. X-axis labels indicate the treatment groups, and the Y-axis, composite IS scores. **C**. Cyst wall damage (DS) scores in cysts. Dots and bars as in B. Panels B and C include cysts from additional infected pigs that were untreated or treated with PZQ in an identical manner as those described in Methods. Treatment groups are indicated in the X-axis labels, and Y-axis shows the composite CD score as described in Methods. IS and CD scores from the untreated (U) and PZQ-treated (P) groups include data from experiments published elsewhere [17]. Treatment groups for B and C are as follows—U: Untreated, P: PZQ, DP: DEX/PZQ, EP: ETN/PZQ. Asterisks indicate statistically significant differences between groups: *- p<0.05; **- p<0.005; ***- p<0.0005; Mann-Whitney U test.

All infected animals had cysts scattered throughout the brain. On gross examination, pericystic capsules were found either to be clear or stained blue due to extravasated EB. As observed previously [22], the proportion of capsules that had EB extravasation was increased 120h after PZQ treatment (Fig 2A; p<0.001, Fisher’s test). The increase in proportion of blue capsules after PZQ treatment was not reduced by pretreatment with DEX or ETN (Fig 2A; p>0.05, Fisher’s test). A total of 339 cysts were collected from all experimental animals, of which 233 cysts with intact pericystic capsules were examined histologically for both IS and DS. To determine if pretreatment with DEX or ETN affected the degree of inflammatory infiltration around cysts or damage to cyst walls, we compared the IS and DS among the experimental groups. No significant differences were noted in the IS or DS in clear capsules (Fig 2B and 2C), likely due to the high variability in the scores. However, IS and DS scores for blue capsules were higher in PZQ-treated pigs compared to untreated pigs. Pretreatment with DEX or ETN reduced the IS of blue cysts compared to PZQ alone (Fig 2B). Analysis of cyst wall damage associated with PZQ treatment revealed that DEX pretreatment, but not pretreatment with ETN, resulted in a decrease in the DS. (Fig 2C) These findings suggest dissociation between the regulation of inflammation by TNF-α, and the induction of cyst wall damage.

### TNFα blockade is associated with inhibition of pro-inflammatory mediators in pericystic capsules

**Fig 3:**
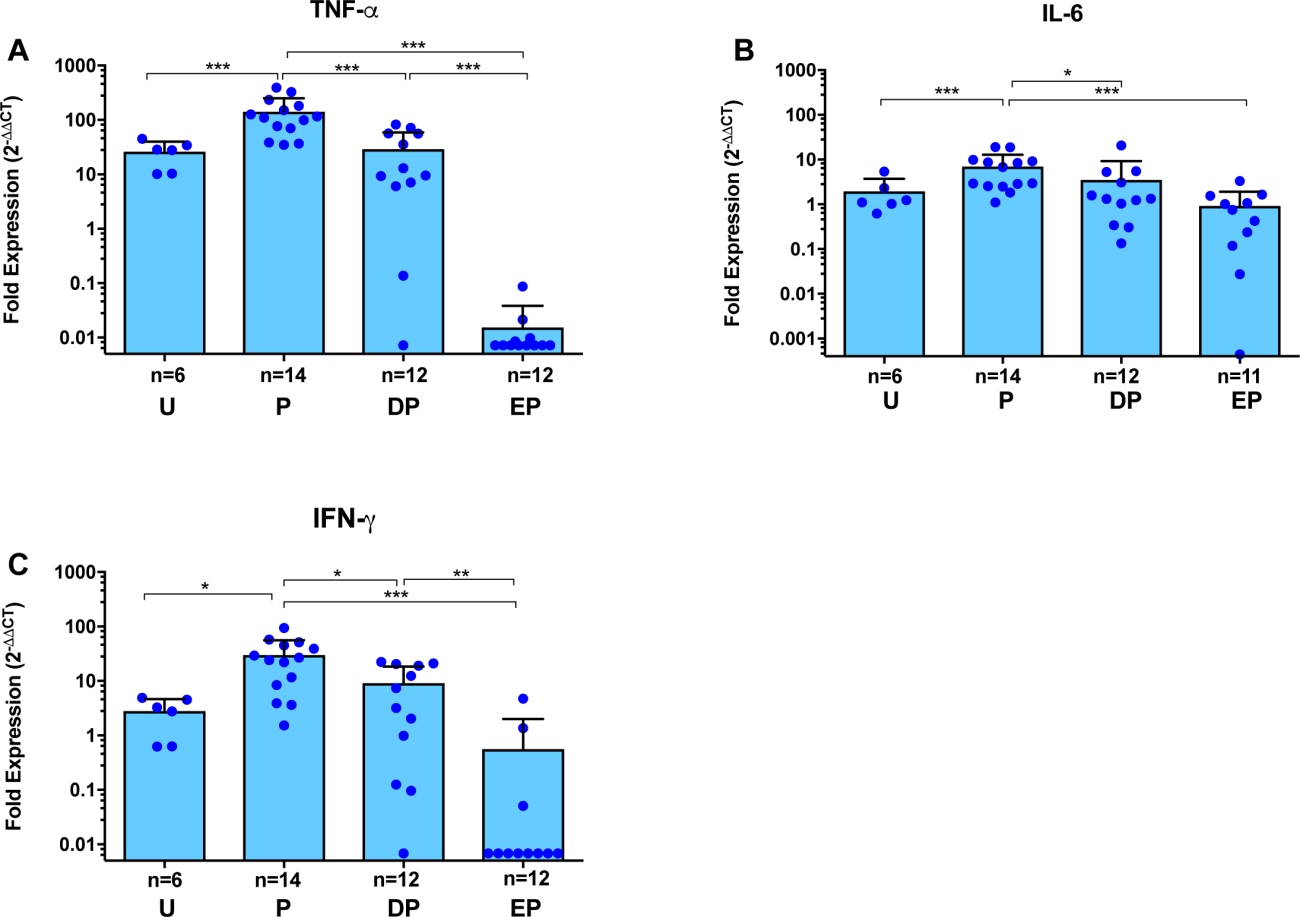
Gene expression analysis for pro-inflammatory cytokines in pericystic brain tissues with Evans blue extravasation, from infected pigs following PZQ treatment with or without pretreatment with anti-inflammatory agents. Expression of each target gene in pericystic tissue from each cyst was determined by quantitative RT-PCR (see Methods). Expression of TNF-α (**A**), IL-6 (**B**) and IFN-γ (**C**) are shown as fold expression normalized first for the housekeeping gene (18s rRNA) and then for uninfected brain tissue. Each dot represents an individual capsule; bars represent the mean and error bars are the standard deviations. Group labels are the same as for Fig 2. Within each study group, the number of cysts differed for TNF-α, IL-6 and IFN-γ. Asterisks indicate statistically significant differences between groups as in Fig 2.

A smaller subset of cysts than those examined histologically was analyzed for expression levels of genes for molecules involved in tissue inflammation (S1 Table). Comparison of three markers of inflammation, TNF-α, IL-6 and IFN-γ, revealed that 120-h after PZQ treatment there was a significant up regulation of all three markers in blue capsules, as expected (Fig 3A–3C, p<0.05 for each). However, TNF-α blockade with ETN prior to PZQ treatment resulted in a profound decrease in the expression of TNF-α compared to PZQ alone (Fig 3A; ~10-fold decrease to untreated baseline, p<0.0005). A smaller, but significant (p<0.005) decrease was observed in DEX pretreated pigs. IL-6 and IFN-γ were similarly inhibited in blue capsules by ETN pretreatment (Fig 3B and 3C). In clear capsules that lack the disruption of vascular integrity, gene expression of pretreatment with ETN or DEX could not be compared to PZQ alone (see S1 Fig). Remarkably, expression of TNF-α was lower in cysts from ETN pretreated pigs than from pigs that did not receive PZQ (Fig 3A; p<0.05, Mann-Whitney U test). There was no significant corresponding inhibition of IFN-γ in the clear cysts (S1 Fig).

### Inhibition of regulatory molecules with TNF-α blockade

**Fig 4:**
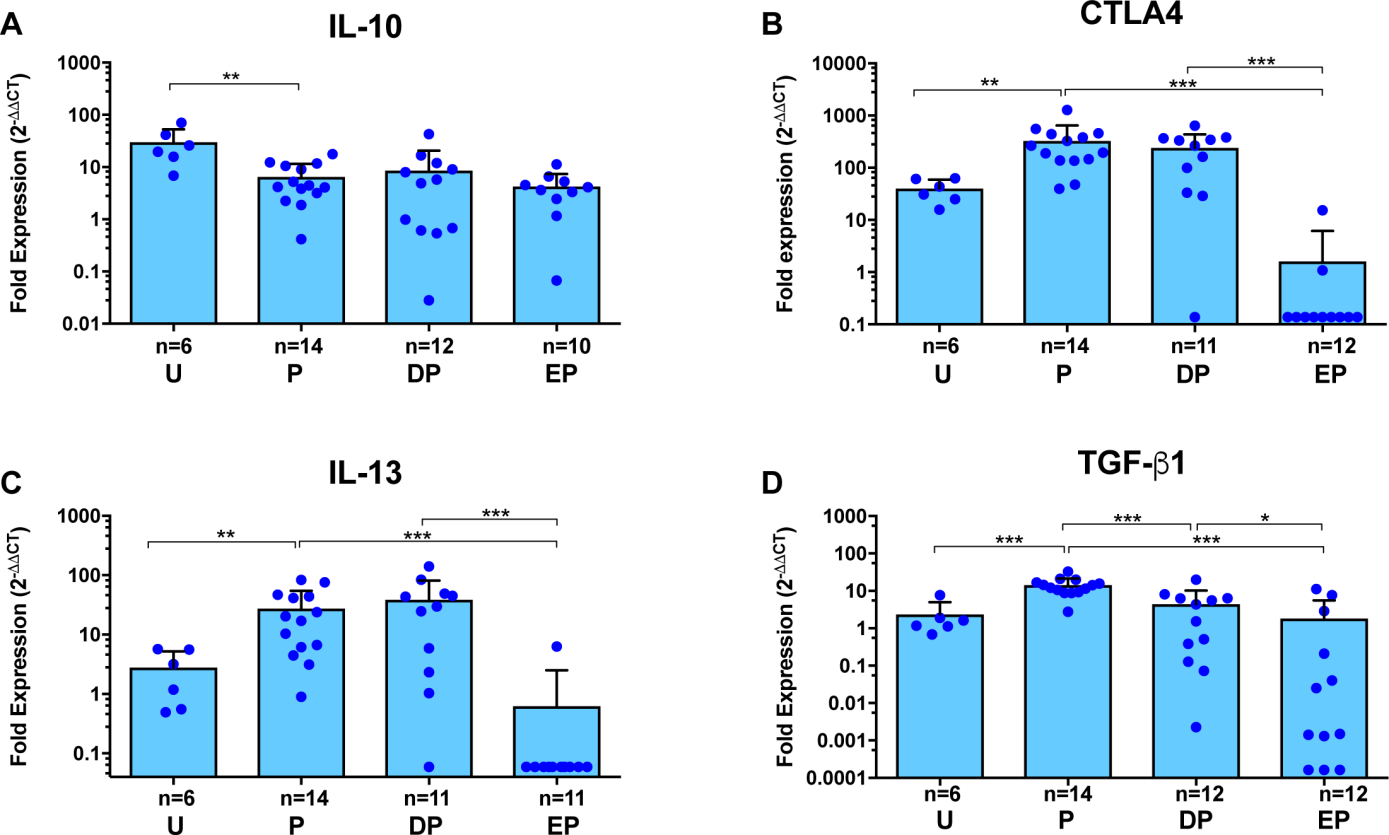
Gene expression analysis for immunoregulatory mediators in pericystic brain tissues with Evans blue extravasation, from infected pigs following PZQ treatment with or without pretreatment with anti-inflammatory agents. Expression of each target gene in pericystic tissues from individual cysts was determined by quantitative RT-PCR (see Methods). Expression of IL-10 (**A**), CTLA4 (**B**), IL-13 (**C**) and TGF-β1 (**D**) are shown as fold expression as detailed in the legend for Fig 3. Each dot represents an individual capsule, bars represent the mean and error bars are the standard deviations. Group labels are the same as for Fig 2 and asterisks indicate level of significance as listed in Fig 2. Within each study group, different numbers of cysts were analyzed for each of the markers shown.

We next analyzed the effect of ETN and DEX pretreatment on counter regulatory pathways. In our previous study, we found variable inhibition of regulatory molecules, so that CD25 and CTLA4 transiently decreased at 48h post PZQ treatment, whereas IL-10 showed persistent decrease 48h post treatment and later [17]. In the present study, IL-10 gene expression was inhibited from baseline after (120-h) PZQ treatment, but neither DEX nor ETN reversed the PZQ-induced inhibition (Fig 4A). In contrast, the expression of three other regulatory molecules, CTLA4, IL-13 and TGF-β, was significantly decreased by ETN (but not by DEX) pretreatment (Fig 4B–4D). While the inhibitory effect of ETN on gene expression of CTLA4 and TGF-β was only apparent around blue cysts, inhibition of IL-13 expression was also significantly decreased around clear cysts in pigs pretreated with ETN compared to DEX (S1 Fig). Unfortunately, there were insufficient numbers of clear cysts in the PZQ alone treated pigs for valid statistical inferences for comparisons with ETN or DEX pretreatment.

### TNF-α blockade inhibits expression of tissue remodeling genes

**Fig 5:**
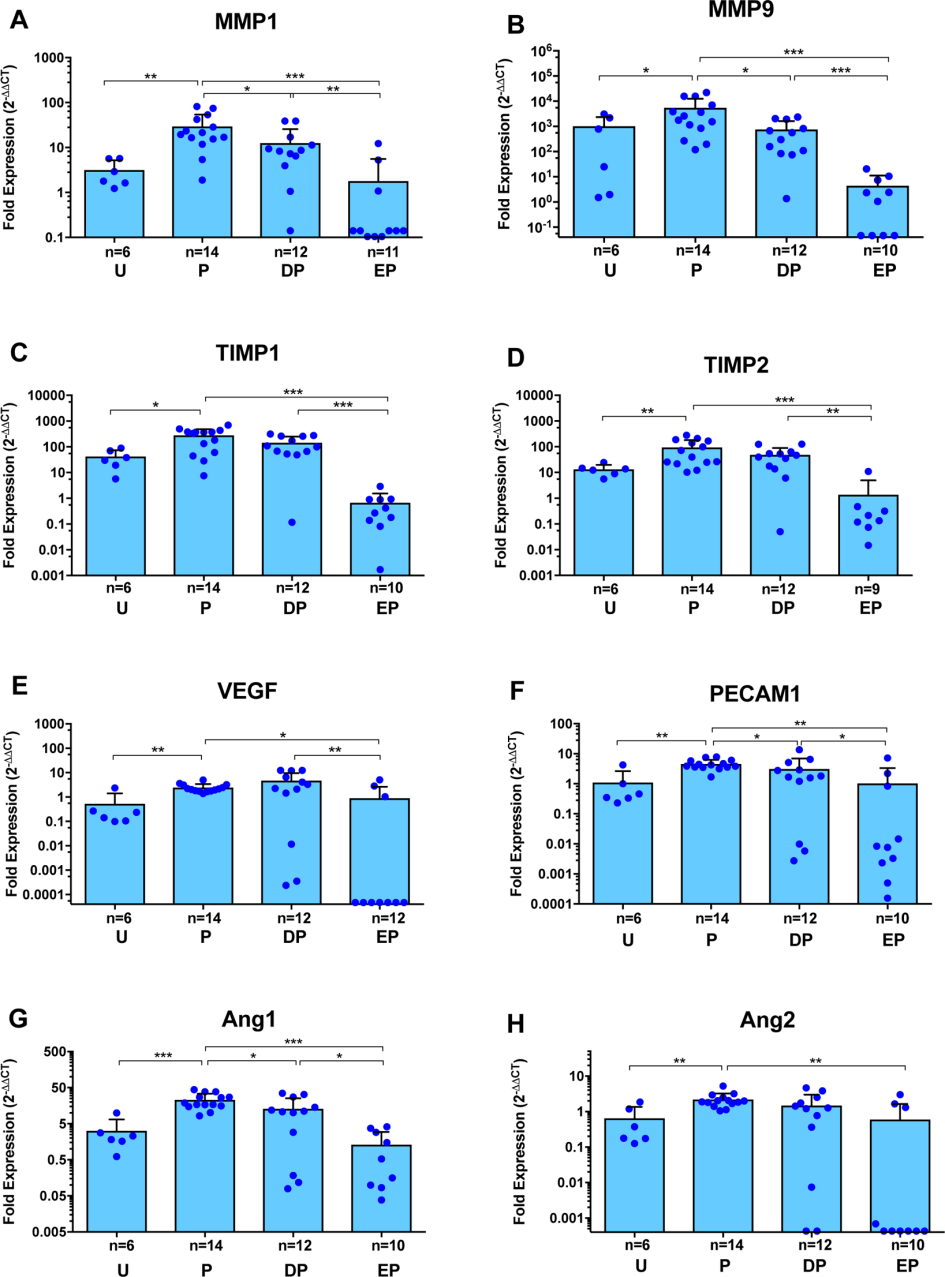
Gene expression analysis for molecules associated with tissue remodeling and endothelial cell functions in pericystic brain tissues with EB extravasation, from infected pigs following PZQ treatment with or without pretreatment with anti-inflammatory agents. Expression of MMP1 (**A**), MMP9 (**B**), TIMP1 (**C**), TIMP2 (**D**), VEGF (**E**), PECAM1 (**F**), angiopoietin 1 (Ang1; **G**) and angiopoietin 2 (Ang2; **H**) are shown as fold expression as detailed in the legend for Fig 3. Each dot represents an individual capsule; bars represent the mean and error bars are the standard deviations. Within each study group, different numbers of cysts were analyzed for each of the markers shown. Group labels are the same as for Fig 2 and asterisks indicate level of significance as listed in Fig 2.

Genes associated with tissue remodeling and also in granuloma formation, such as matrix metalloprotease (MMP) 2 and MMP9, as well as their regulators, the tissue inhibitors of metalloproteases (TIMP) 1 and TIMP2, are up regulated following PZQ treatment in rodent models of NCC [23, 24] and in infected pigs [17]. Analysis of these genes in the present study revealed profound down regulation of MMP2, MMP9, TIMP1 and TIMP2 in blue cyst capsules from ETN-pretreated pigs compared to pigs who received PZQ alone (Fig 5A–5D). These observations are consistent with the global inhibition of gene expression that appears to accompany TNF-α blockade. Interestingly, DEX pretreatment did not significantly down regulate transcripts that were markedly inhibited by the TNF-α blockade.

### Inhibition of the TNF-α pathway impairs expression of genes regulating endothelial cell functions

Expression of genes involved in endothelial activation such as PECAM1, and angiogenesis such as VEGF, angiopoietin 1 and 2 (Fig 5E–5H) are commonly involved in the pathogenesis of parasitic infections [25–27]. Our prior findings that showed increased vascular leakage in the cyst capsules following PZQ treatment of pigs [17, 18] also suggest that endothelial integrity and function may be involved in the resulting inflammatory pathology. We found that ETN pretreatment resulted in a significant inhibition of transcription of all these molecules, which were up regulated by PZQ treatment alone. Taken together, these data reveal that TNF-α blockade resulted in transcription inhibition of a diverse range of inflammatory and regulatory pathway molecules and suggest an important role for TNF-α in regulating the inflammation that predictably follows PZQ treatment.

**Fig 6:**
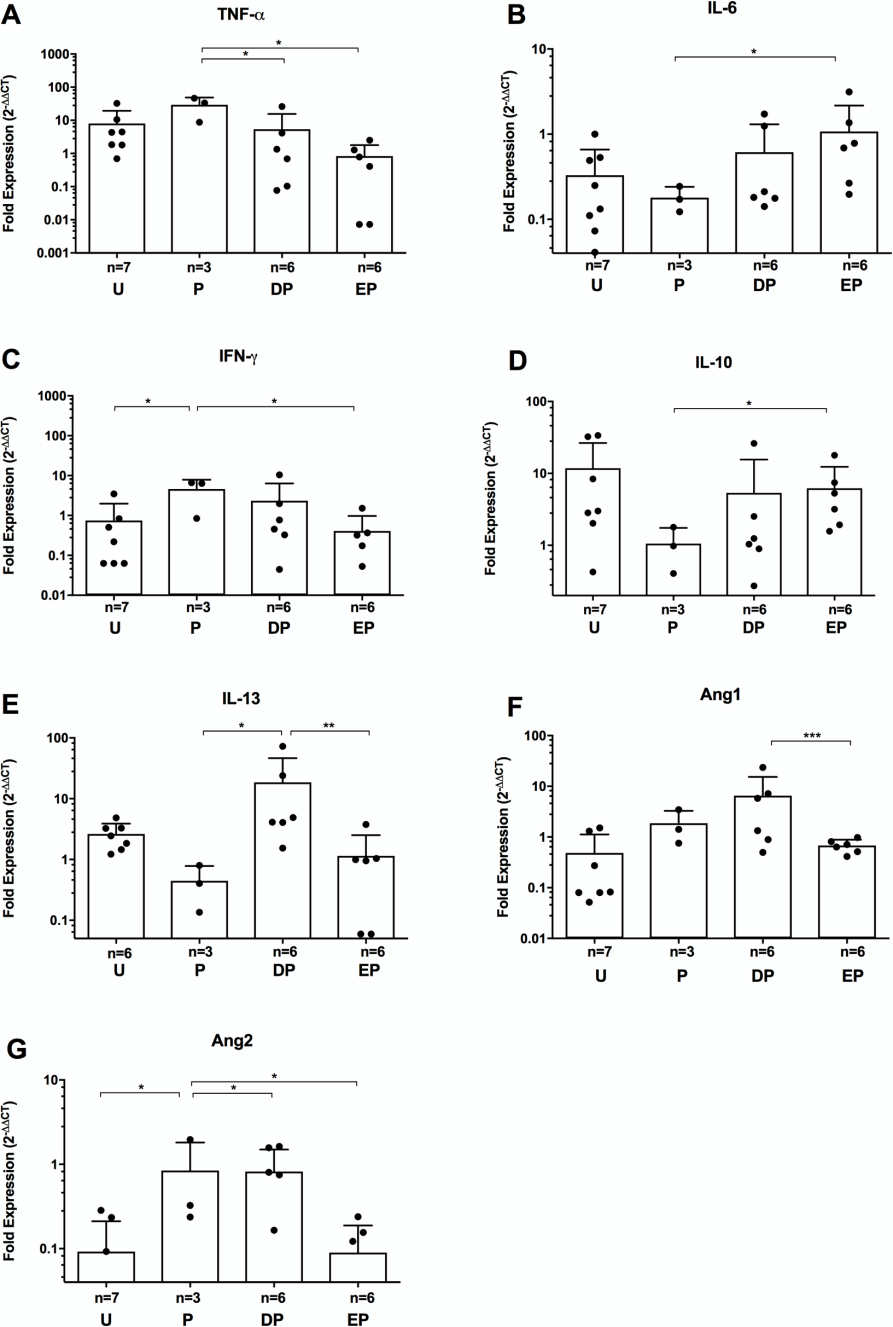
Gene expression patterns in pericystic tissues lacking Evans blue extravasation. Expression of mRNA for the target genes tested for blue cysts that showed statistically significant differences between PZQ-treated and ETN- or DEX-pretreated pigs were evaluated for clear cysts by quantitative RT-PCR (as described in the legend for Fig 3 and in Methods). The target genes are: TNF-α (**A**), IL-6 (**B**), IFN-γ (**C**), IL-10 (**D**), IL-13 (**E**), Ang-1 (**F**), and Ang-2 (**G**). There were no statistically significant differences between the clear cysts among the experimental groups for TGF-β1, VEGF, MMP1, MMP9, TIMP1, TIMP2 or PECAM1 (S1 Fig). Within each study group the numbers of cysts analyzed differed for each of the markers shown. Group labels are the same as for Fig 2 and asterisks indicate level of significance as listed in Fig 3.

We also determined gene expression levels for the genes discussed above in tissues around cysts from the four experimental groups that did not have EB leakage and, therefore, were not demonstrating disruption of the BBB (Fig 6), referred to as “clear” cysts. The total number of clear cysts was low because the PZQ treatment, which the three experimental groups received, appears to induce BBB leakage in the large majority of cysts. Although there were decreases in gene expression of some inflammatory markers (e.g., TNF-α, IFN-γ) in clear cysts from DEX and ETN-treated pigs that were similar to those seen in blue cysts, other inflammatory markers showed increased expression in DEX- and ETN-treated pigs (e.g., IFN-γ and Ang2; Fig 6C and 6G). These differences between the blue and clear cysts probably reflect the lower level of tissue penetration of ETN in the latter due to an intact BBB, that may influence it’s effectiveness in the tissues.

## Discussion

In *T*. *solium*-infected humans and pigs, inflammation around dying cysts occurs frequently and predictably within a week of cysticidal treatment in humans and in animal models of NCC [28–33]. In humans, the evidence for this manifest as an increased incidence of headaches, seizures and other neurologically based symptoms associated with the development of gadolinium enhancement and edema around some cysts seen on MRI imaging within the first week after initiation of cysticidal treatment. Limited histopathological examination of brain tissues around degenerating cysts in patients post-treatment or with untreated “degenerated” cysts that shows infiltration of inflammatory cells [5] additionally supports the concept of treatment induced inflammation. Previous studies performed by ourselves and others demonstrated similar post-treatment inflammatory reactions in pigs [17, 18, 22, 33–35]. In a practical sense, these detrimental side effects of antiparasitic treatment complicate medical treatment of NCC because they are a cause of morbidity and need to be prevented and controlled with corticosteroids, the use of which is associated with a variety of side effects [1, 5, 36, 37]. In a review focused on murine models of cysticercosis, one author discussed the concept of using approaches other than corticosteroids to inhibit inflammation associated with cysticidal treatment [38].

In the CNS, TNF-α produced by migrated peripheral immune cells or by microglia and astrocytes in the presence of inflammation [39, 40] plays an important role in inducing and maintaining inflammation that occurs in NCC. Evidence for this includes high levels of TNF-α reported in CSF samples from patients [12, 41–47] and increased expression of the TNF-α gene in pericystic capsules in pigs treated with PZQ [17, 48, 49]. TNF-α has also been shown to be a key cytokine in maintaining inflammation in other inflammatory diseases involving the CNS, such as some causes of meningitis and autoimmune encephalitis [50–56]. Therefore, we hypothesized that blockade of TNF-α should mitigate post-treatment inflammation in NCC. Our data show that TNF-α blockade during PZQ treatment resulted in a broad and unique pattern of inhibition of gene expression for inflammation promoting proteins, regulating molecules, a number of pathways of tissue remodeling substances and molecules that modulate endothelial cellular function. Notably, not all the genes tested were inhibited by ETN: for example, IL-10 expression in PZQ plus ETN treated pigs did not differ significantly from pigs receiving PZQ alone or PZQ plus DEX. In contrast, DEX administration prior to PZQ treatment significantly inhibited only TNF-α, IL-6, IFN-γ, TGF-β and Ang1 relative to PZQ treatment alone (Figs 3, 4 and 6). The lower anti-inflammatory responses to DEX compared to ETN was surprising, since corticosteroids are known for their potent and global immunosuppressive activity [57–59]. However, pigs are known to be relatively insensitive to the immunosuppressive effects of corticosteroids [60]. The effectiveness of ETN-mediated inhibition of multiple pathways of inflammation and tissue remodeling that is demonstrated by these data suggest a significant role for TNF-α in post-PZQ inflammatory responses

An interesting finding in this study was an apparent dissociation between the effects of ETN on gene expression for inflammatory mediators and regulators (Figs 3 and 4) and its effect on cellular infiltration in pericystic tissues (Fig 2B). TNF-α triggers a cascade of inflammatory cytokines, but also promotes endothelial cell contribution to local inflammation via the display of different combinations of adhesion molecules for leukocytes, including E-selectin, intercellular adhesion molecule-1 (ICAM-1) and vascular cell adhesion molecule-1 (VCAM-1) in a distinct temporal, spatial and anatomical pattern [61, 62]. In combination with the release of chemokines (including IL-8, MCP-1 and CCL2) [63], these responses lead to recruitment of different populations of leukocytes, so blockade of TNF-α would normally be expected to inhibit cellular recruitment. However, our data (Fig 2B) reveal a weak, albeit significant, reduction in scores signifying only a small decrease in cellular infiltration. The reason for this apparent dissociation in the two functional properties of TNF-α in this model is unclear, and may relate to our use of a human TNF-α blocker in pigs or possibly a differential effect of TNF-α concentration on the two processes. Interestingly, the effect of TNF-α blockade on parasite damage, as reflected in the cyst wall damage scores (Fig 2C), suggests that the inhibition of measured proinflammatory, regulatory and other molecules did not inhibit damage to cysts caused by PZQ, as was found with DEX pretreatment.

ETN, a licensed biologic, has been used for TNF-α blockade for over 20 years [64, 65] and has shown remarkable efficacy as an anti-inflammatory agent in rheumatoid arthritis and inflammatory bowel diseases [62, 66]; its safety profile is well known. Our data demonstrate that TNF-α blockade induces potent suppression of post-treatment pericystic inflammation in a natural infection model of NCC. The inhibitory effect of TNF-α in this model was comparable to that of DEX, a potent inhibitor of inflammation in many settings. This study provides proof of principle that TNF-α blockade, used alone or as a steroid-sparing agent, may be a viable strategy for management of post-PZQ pericystic inflammation.

## Supporting information

S1 Table Characteristics of cysts subjected to histology and quantitative PCR analysis. (DOC)

S2 Table Summary of cysts demonstrating disruption of vascular integrity (blue capsules) in the study groups. (DOC)

S1 Fig Gene expression patterns in pericystic tissues lacking Evans blue extravasation. Expression of mRNA for the target genes tested for blue cysts were evaluated for clear cysts by quantitative RT-PCR (as described in the legend for Fig 3 and in Methods). Expression levels of TGF-β1 (A), VEGF (B), MMP1 (C), MMP9 (D), TIMP1 (E), TIMP2 (F) and PECAM1 (G) did not differ significantly among the experimental groups. Within each study group the numbers of cysts analyzed differed for each of the markers shown. Group labels are the same as for Fig 3 and asterisks indicate level of significance as listed in Fig 3.
(TIFF)
