# Supplementary material for: TNF-α blockade suppresses pericystic inflammation following anthelmintic treatment in porcine neurocysticercosis
Source: PLoS Negl Trop Dis. 2017 Nov 30;11(11):e0006059. doi: 10.1371/journal.pntd.0006059 (PMC5708608; doi:10.1371/journal.pntd.0006059)
Supplement: S1 Table — (DOC) [file pntd.0006059.s001.doc]

**S1 Table. Characteristics of cysts subjected to** histology and quantitative PCR analysis

|  |  | **Histology** |  | **qPCR** | | |
| --- | --- | --- | --- | --- | --- | --- |
| **Condition** | **clear** | **blue** | **total** | **clear** | **blue** | **total** |
| **Untreated (U)** | 14 | 33 | 47 | 7 | 6 | 13 |
| **PZQ (P)** | 7 | 47 | 54 | 3 | 14 | 17 |
| **DEX/PZQ (DP)** | 15 | 56 | 71 | 6 | 12 | 18 |
| **ETN/PZQ (EP)** | 16 | 45 | 61 | 6 | 12 | 18 |
| **Total** | **52** | **181** | **233** | **22** | **44** | **66** |
