# Supplementary material for: TNF-α blockade suppresses pericystic inflammation following anthelmintic treatment in porcine neurocysticercosis
Source: PLoS Negl Trop Dis. 2017 Nov 30;11(11):e0006059. doi: 10.1371/journal.pntd.0006059 (PMC5708608; doi:10.1371/journal.pntd.0006059)
Supplement: S2 Table — (DOC) [file pntd.0006059.s002.doc]

| **S2 Table. Summary of cysts collected from each study group** | | | | | | | | | | | |  |
| --- | --- | --- | --- | --- | --- | --- | --- | --- | --- | --- | --- | --- |
|  |  | **per animal** | | | | | **average (per group)** | | | | |  |
|  | **Pigs (n)** | **clear** | | **blue** | | **blue/clear** | **clear** | | **blue** | | **blue/clear** | |
| **Untreated** | 3 | 13 | 25 | | 1.92 | | 4.7 ± 7.2 | 11 ± 12.2 | | 3.5 ± 2.2 | |  |
| **(U)** |  | 1 | 5 | | 5.00 | |  |  | |  | |  |
|  |  | 0 | 3 | | indeterminate | |  |  | |  | |  |
| **PZQ** | 9 | 0 | 5 | | indeterminate | | 1.3 ± 1.9 | 8.6 ± 10.7 | | 5.2 ± 2.9 | |  |
| **(P)** |  | 0 | 1 | | indeterminate | |  |  | |  | |  |
|  |  | 6 | 32 | | 5.33 | |  |  | |  | |  |
|  |  | 0 | 2 | | indeterminate | |  |  | |  | |  |
|  |  | 2 | 12 | | 6.00 | |  |  | |  | |  |
|  |  | 2 | 19 | | 9.50 | |  |  | |  | |  |
|  |  | 0 | 1 | | indeterminate | |  |  | |  | |  |
|  |  | 1 | 3 | | 3.00 | |  |  | |  | |  |
|  |  | 1 | 2 | | 2.00 | |  |  | |  | |  |
| **DEX+PZQ** | 6 | 0 | 10 | | indeterminate | | 2.8 ± 4.1 | 11.8 ± 10.8 | | 3.3 ± 0.6 | |  |
| **(DP)** |  | 9 | 30 | | 3.33 | |  |  | |  | |  |
|  |  | 0 | 7 | | indeterminate | |  |  | |  | |  |
|  |  | 7 | 19 | | 2.71 | |  |  | |  | |  |
|  |  | 0 | 1 | | indeterminate | |  |  | |  | |  |
|  |  | 1 | 4 | | 4.00 | |  |  | |  | |  |
| **ETN+PZQ** | 6 | 2 | 15 | | 7.50 | | 4.2 ± 5.5 | 15 ± 12.8 | | 4.9 ± 1.9 | |  |
| **(EP)** |  | 0 | 0 | | indeterminate | |  |  | |  | |  |
|  |  | 3 | 17 | | 5.67 | |  |  | |  | |  |
|  |  | 1 | 5 | | 5.00 | |  |  | |  | |  |
|  |  | 4 | 16 | | 4.00 | |  |  | |  | |  |
|  |  | 15 | 37 | | 2.47 | |  |  | |  | |  |
|  | **Total** | **68** | **271** | | **3.99** | |  |  | |  | |  |
