# Supplementary figures and images for: TNF-α blockade suppresses pericystic inflammation following anthelmintic treatment in porcine neurocysticercosis
Source: PLoS Negl Trop Dis. 2017 Nov 30;11(11):e0006059. doi: 10.1371/journal.pntd.0006059 (PMC5708608; doi:10.1371/journal.pntd.0006059)

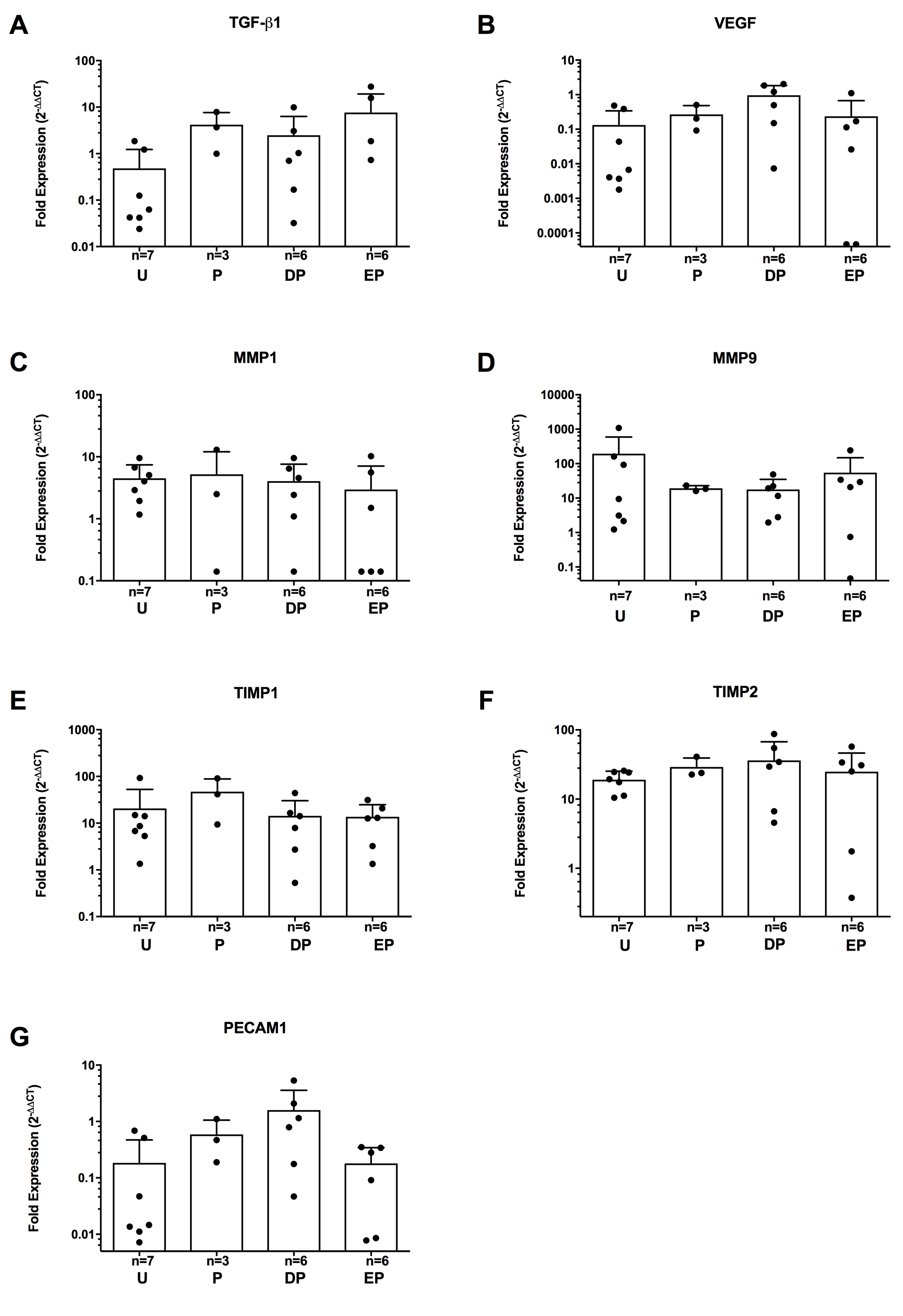

Supplement: S1 Fig — Expression of mRNA for the target genes tested for blue cysts were evaluated for clear cysts by quantitative RT-PCR (as described in the legend for Fig 3 and in Methods). Expression levels of TGF-β1 (A), VEGF (B), MMP1 (C), MMP9 (D), TIMP1 (E), TIMP2 (F) and PECAM1 (G) did not differ significantly among the experimental groups. Within each study group the numbers of cysts analyzed differed for each of the markers shown. Group labels are the same as for Fig 3 and asterisks indicate level of significance as listed in Fig 3. (TIFF) [file pntd.0006059.s003.tiff]
